# Exploring Racial and Ethnic Differences in Utilization of Medications for Obesity Management in a Nationally Representative Survey

**DOI:** 10.1007/s40615-024-02248-x

**Published:** 2024-12-17

**Authors:** Kimberly Narain, Christopher Scannell

**Affiliations:** 1 UCLA Division of General Internal Medicine and Health Services, Research (GIM/HSR), 1100 Glendon Ave., Suite 850, Los Angeles, CA 90024 USA; 2 UCLA Iris Cantor Women’s Health Center, University of California, Los Angeles, CA USA; 3 University of Southern California Schaeffer Institute, University of Southern California, Los Angeles, CA USA; 4 VA Greater Los Angeles Healthcare System, Los Angeles, CA USA

**Keywords:** Obesity, Disparities, Medications, Race, Ethnicity

## Abstract

**Background:**

The burden of obesity falls disproportionately on some racial and ethnic minority groups.

**Objective:**

To assess for racial and ethnic differences in the utilization of obesity-management medications among clinically eligible individuals.

**Design:**

Medical Expenditure Panel Survey (2011–2016, 2018 and 2020) data and a cross-sectional study design was used to assess for racial and ethnic differences in obesity-management medication utilization. Descriptive statistics and multivariable logistic regression models were used to identify the association of race and ethnicity with obesity-management medication utilization. Adjusted models controlled for demographics, socioeconomic status, obesity class, diabetes status, number of chronic conditions, insurance status, and geographic region.

**Participants:**

Adults with a BMI ≥ 30 kg/m^2^ and individuals with a BMI ≥ 27 kg/m^2^ with ≥ 1 weight-related condition.

**Main Measures:**

The primary outcome measure was utilization of an FDA-approved medication for obesity-management during the study period. The primary independent predictor was race and ethnicity. Separate indicator variables were created for each racial and ethnic group (Non-Hispanic Asian, Non-Hispanic Black, Hispanic, and Non-Hispanic White (reference group)).

**Key Results:**

In adjusted analyses, Asian (aOR, 0.36; 95% CI, 0.16 to 0.77; *P* < 0.01), Black (aOR, 0.51; 95% CI, 0.39 to 0.68; *P* < 0.001) and Hispanic individuals (aOR, 0.70; 95% CI, 0.49 to 0.98; *P* = 0.04) had significantly lower odds of utilizing obesity-management medications compared to White individuals.

**Conclusions:**

The results of this study suggest that there are racial and ethnic disparities in the use of obesity-management medications.

**Supplementary Information:**

The online version contains supplementary material available at 10.1007/s40615-024-02248-x.

## Introduction

Obesity, defined as a Body Mass Index (BMI) ≥ 30 kg/m^2^, is associated with a myriad of chronic health conditions [1]. It is an important driver of premature mortality in the United States, with an estimated 1,300 deaths per day (500,000 deaths annually) being linked to excess weight [2]. The burden of obesity falls disproportionately on some racial and ethnic minority groups. According to the Centers for Disease Control and Prevention, the prevalence of obesity among adults, based on BMI, is 16.1%, 49.9%, 45.6% and 41.4% for Non-Hispanic Asian (hereafter, Asian), Non-Hispanic Black (hereafter, Black), Hispanic, and Non-Hispanic White (hereafter, White) individuals, respectively [3].

Sustained weight loss of 5% to 15% is advised to improve many health conditions associated with obesity [4]. The clinical guidelines of several professional societies such as the American Association of Clinical Endocrinologists and American College of Endocrinology recommend the use of adjunctive pharmacotherapy to help achieve this goal if individuals are unable to do so with lifestyle modification alone [4]. Weight loss with obesity management medications has typically ranged from 6%−12% of total body weight [5]. However, the recently FDA-approved glucose-dependent insulinotropic polypeptide (GIP) and glucagon-like peptide-1 (GLP-1) receptor agonist tirzepatide has been linked with more than 20% body weight loss in a randomized controlled trial and even more potent drug classes are on the way [6, 7]. Consequently, pharmacotherapy in combination with lifestyle modification has the potential to turn the tide on the obesity epidemic.

There is reason to believe that there are racial and ethnic differences in access to medications for obesity management. Healthcare access barriers, socioeconomic factors, knowledge differences, weight-related, and racial bias from physicians and mistrust of the healthcare delivery system among patients may have a disproportionate impact on the utilization of obesity-management medications among individuals from racial and ethnic minority groups, relative to White individuals [8–10]. Furthermore, there may be cultural differences that produce differences in the demand for obesity-management medications across racial and ethnic lines. For example, some racial and ethnic groups have standards of beauty that place value on larger body types [11].

Prior studies evaluating obesity-management medication utilization have produced mixed results regarding racial and ethnic differences in utilization. These differences likely stem from the use of different data sets, years of data, study populations and covariates. Limitations of the extant literature include the use of relatively old data in most studies, the inclusion of a limited number of racial and ethnic minority groups, and lack of control for potential confounders such as education and income [12–17].

Using Medical Expenditure Panel Survey (MEPS) data from 2002–2007 Mehta et al. (2013) documented lower utilization of obesity-management medication among Black but not Hispanic individuals [15] MacEwan et al. (2021) addressed this question using later years of MEPS data (2015–2018) and did not find any significant differences in obesity-management medication utilization between White individuals and individuals from racial and ethnic minority groups (Black, other race, Other Hispanic, Mexican American) [13]. However, MacEwan et al. did not control for potential confounders such as education, income, and obesity class. Claridy et al. (2021) examined this question among a population of individuals with obesity and at least one physician visit. In this population receiving health care, there were no differences between White individuals and individuals from racial and ethnic minority groups, who were aggregated into a single group [10]. Gasoyan et al. (2023) addressed this question using electronic health record data from Ohio and Florida (2015–2023) and found that being Black, other race and Hispanic was negatively associated with receiving an obesity-management medication [18]. Lastly, Saxon et al. (2019) documented the prevalence of obesity-management medication utilization among eligible individuals from several different racial and ethnic groups (American Indian/Alaskan Native, Asian, Black, Hispanic, Native Hawaiian or Pacific Islander other) receiving care in any one of eight large healthcare systems [17]. While the proportion of obesity management medication utilization seemed to be lower among Asian and Hispanic individuals, relative to White individuals, the unadjusted nature of the analysis precludes any definitive conclusions from being drawn regarding racial and ethnic differences in obesity-management medication utilization. Of note, Asian-specific BMI cutoffs (Overweight = BMI 23–26.99 kg/m^2^ & Obesity = BMI ≥ 27.5 kg/m^2^) were not used in this study, potentially leading to an overestimation of the prevalence of obesity-management medication use among Asian individuals [19].

Exploring the relationship between race and ethnicity and obesity-management medication use is a fundamental step in ensuring equitable access to medications that have the potential to turn the current trajectory of obesity and obesity-related diseases in conjunction with lifestyle modification. As such, we build on this prior work by including more recent years of MEPS data, by considering utilization of several different racial and ethnic groups (Asian, Black, Hispanic, White), and by considering a range of socioeconomic variables as potential confounders to assess the relationship between race, ethnicity, and obesity-management medication use. Furthermore**,** we conduct sensitivity analyses assessing racial and ethnic differences in obesity-management medication utilization using Asian-specific BMI cutoffs for Asian individuals, stratifying the study population across obesity category, and considering the use of off-label medications for obesity management. We hypothesize that there will be racial and ethnic differences in obesity-management medication utilization that will persist despite controlling for a wide range of potential confounders.

## Methods

The study protocol was exempted from review by the University of California Los Angeles Institutional Review Board. This study followed the Strengthening the Reporting of Observational Studies in Epidemiology (STROBE) reporting guideline for cross-sectional studies.

### Study Data

The “Household Component” of the MEPS survey from years 2011 to 2020 was used with the exception of the 2017 and 2019, years which excludes BMI data due to a survey change that only allows for BMI collection in even years. MEPS draws from a nationally representative subsample of households that participated in the prior year's National Health Interview Survey. It provides de-identified data from individual households and their members, which is supplemented by data from their medical providers.

### Study Sample

The study population for the main analysis was limited to individuals with a clinical indication for utilization of an obesity-management medication, specifically individuals meeting criteria for obesity by BMI (BMI ≥ 30 kg/m^2^) and individuals with an elevated BMI (BMI ≥ 27 kg/m^2^) and at least one weight-related condition (hypertension, hypercholesterolemia, coronary artery disease, angina, stroke, myocardial infarction, diabetes, asthma, arthritis, history of joint pain, and obesity-related cancer) [13]. MEPS provides a continuous variable for BMI for adults based off self-reported height and weight that ranges from 10–50 kg/m^2^. The race and ethnicity variables were constructed from a categorical variable based on self-reported race and ethnicity that was obtained from the interview respondent during the initial MEPS interview. The race and ethnicity variable provided by MEPS has five responses (1. Hispanic 2. Non-Hispanic Asian 3. Non-Hispanic Black, 4. Non-Hispanic “other race” or multiple races, and 5. Non-Hispanic White). Separate indicator variables for each racial and ethnic group were created. White was the reference group. The study population excluded individuals identified as “Non-Hispanic other race or multiple races” due to the inability to theorize about the factors underlying any observed differences in obesity-management medication utilization among this particular subgroup of unknown racial and ethnic composition.

The outcome of interest, obesity-management medication utilization, was constructed using an indicator variable that was coded as “1” if the respondent had been prescribed an FDA-approved medication for obesity management at any time during the prior year and coded as “0” otherwise. In line with the work of MacEwan et al., buproprion/naltrexone, diethylpropion, liraglutide, lorcaserin, orlistat, phendimetrazine, phentermine, and phentermine/topiramate were considered obesity-management medications [13].

Adjusted analyses controlled for several factors that might be associated with race and ethnicity as well as obesity-management medication utilization including demographics (age category, gender, and marital status), socioeconomic status (education, employment, and family income), number of chronic diseases (0–1 = reference since all individuals classified as overweight included in the study sample will have at least one chronic disease by definition, ≥ 2), the presence of diabetes (reference = no diabetes), weight category (overweight + ≥ 1 weight-related condition = reference, obesity class 1 (BMI = 30–34.9 kg/m^2^), class 2 (BMI = 35–39.9 kg/m^2^), class 3 (BMI = 40–49.9), class 4 (BMI = 50) [20], health insurance (private = reference, Medicaid, Medicare, other, uninsured), geographic region (Northeast = reference, Midwest, South, and West), and year fixed-effects to control for clustering of data within years and non-observable year-specific variation across the study population [21, 22].

## Statistical Analysis

Descriptive statistics were used to calculate the proportion of the eligible population using an obesity-management medication across racial and ethnic groups. The adjusted Wald test was used on survey-weighted estimates to compare utilization across racial and ethnic minority subgroups to that of White individuals [23]. Additionally, logistic regression models were run to assess the adjusted relationship between race and ethnicity and obesity-management medication utilization. All models were weighted using MEPS survey weights. The point estimates for adjusted models were odds ratios. A *P*-value of < 0.05 was considered statistically significant for all estimates. For each model, we implemented an established strategy to adjust the BMI measure for reporting bias [24]. Missing data was dealt with using complete case analysis.

In the sensitivity analysis including Asian-specific BMI thresholds, the study population was expanded to include individuals meeting criteria for obesity by BMI (BMI ≥ 30 kg/m^2^ and BMI ≥ 27 kg/m^2^ if Asian) and individuals with an elevated BMI (BMI ≥ 27 kg/m^2^ and BMI = 23–26.9 kg/m^2^ if Asian) and at least one weight-related condition. Additionally, a set of sensitivity analyses stratifying individuals across weight category (overweight + ≥ 1 obesity-related co-morbidity, Class 1 obesity and Class 2 obesity or higher) was run. Lastly, a sensitivity analysis was run considering some medications for potential off-label as obesity-management medications [25]. Specifically, this iteration of the obesity-management medication utilization measure combined the original measure with off-label GLP-1 receptor agonist and separate components of combination pills prescribed within the same year. GLP-1 receptor agonists were included based on studies documenting increased off-label use of these medications for obesity-management [26]. Individual components of combination pills prescribed in the same year were included in the outcome due to the decreased likelihood of patients having separate indications for each of these medications in the same year, relative to the medications being used for obesity-management.

## Results

**Fig. 1:**
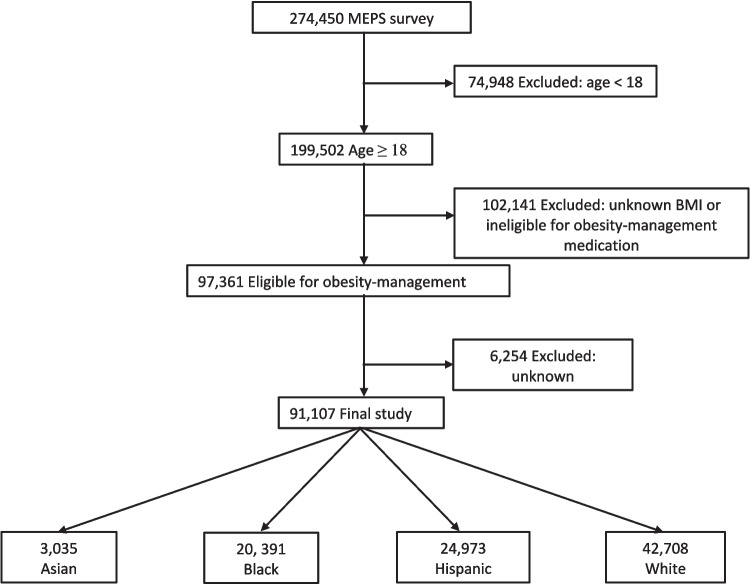
Sample Size Flow Chart

The study sample consisted of 91,107 adults aged 18 years or older who were eligible for obesity-management medications (Fig. 1). Among them, 63,811 individuals (weighted percentage, 67.9%) were classified as obese and 27,296 were classified as overweight with at least one weight-related condition (32.1%). Analyzing the sample by race and ethnicity revealed that 3,035 individuals (2.7%) were classified as Asian, 20,391 (14.0%) were classified as Black, 24,973 (15.7%) were classified as Hispanic, and 42,708 (67.6%) were classified as White. Notable demographic and health differences between the individual subgroups included Asian, Black, and Hispanic individuals being younger but less likely to have two or more weight-related comorbidities compared to White individuals (Table 1). Additionally, the proportion of Asian individuals was more evenly split between those with obesity (46.8%) and those who were overweight with one weight-related condition (53.2%) compared to other subgroups, which predominantly had obesity. Furthermore, the prevalence of diabetes was 19.8%, 18.6%, 16.0% and 15.0% among Asian, Black, Hispanic, and White individuals, respectively. Other demographic characteristics demonstrated heterogeneity across the individual subgroups. Specifically, with respect to socioeconomic status, the proportion of individuals with some college education (64.9%, 42.8%, 31.1% and 54.0%), family income ≥ 400% of the Federal Poverty Level (50.3%, 27.1%, 22.7% and 47.2%), and private health insurance coverage (58.4, 41.5, 36.7 and 52.9) were quite divergent across Asian, Black, Hispanic and White individuals, respectively.

**Table 1:** Obesity Management Medication Utilization and Demographic Characteristics by Race & Ethnicity Among Medication-Eligible 18 + Year-Olds

	Asian	Black	Hispanic	White	*P*-value
Total, No	3035	20,391	24,973	42,708	N/A
Population Weighted, No	927,243	15,225,433	17,041,473	73,272,023	N/A
Obesity-Management Medication Utilization, %	0.34 (0.17 to 0.68)	0.81 (0.65 to 1.00)	0.74 (0.57 to 0.95)	1.20 (1.04 to 1.39)	< 0.001
Diagnosis, %					
Overweight + Weight-Related Condition	53.2 (50.6 to 55.7)	23.7 (22.8 to 24.7)	27.9 (26.9 to 28.8)	34.0 (33.2 to 34.9)	< 0.001
Obesity Class 1	38.0 (35.6 to 40.5)	42.6 (41.6 to 43.6)	47.8 (47.0 to 48.7)	41.9 (41.2 to 42.6)	< 0.001
Obesity Class 2	7.0 (5.8 to 8.5)	19.3 (18.5 to 20.2)	16.0 (15.3 to 16.7)	15.2 (14.6 to 15.7)	< 0.001
Obesity Class 3	1.6 (1.1 to 2.4)	11.8 (11.1 to 12.5)	7.2 (6.7 to 7.7)	7.7 (7.3 to 8.2)	< 0.001
Obesity Class 4	0.2 (0.1 to 0.6)	2.5 (2.2 to 2.8)	1.1 (0.9 to 1.3)	1.2 (1.1 to 1.4)	< 0.001
Age, years	49.9 (48.7 to 51.1)	48.1 (47.6 to 48.6)	45.2 (44.8 to 45.6)	53.3 (52.9 to 53.7)	< 0.001
Age, %					
18–34 years	20.2 (17.7 to 23.0)	24.3 (23.3 to 25.4)	28.8 (27.8 to 29.8)	16.3 (15.5 to 17.0)	< 0.001
35–54 years	41.3 (38.4 to 44.2)	39.4 (38.3 to 40.5)	43.7 (42.7 to 44.8)	34.2 (33.3 to 35.2)	< 0.001
55–64 years	17.9 (16.0 to 20.1)	19.3 (18.4 to 20.2)	14.7 (13.9 to 15.5)	22.3 (21.6 to 23.0)	< 0.001
65–74 years	13.5 (11.7 to 15.5)	11.2 (10.4 to 12.0)	8.5 (7.9 to 9.2)	16.7 (16.1 to 17.4)	< 0.001
75 + years	7.1 (5.7 to 8.8)	5.8 (5.3 to 6.4)	4.3 (3.9 to 4.8)	10.5 (9.9 to 11.1)	< 0.001
Female, %	49.3 (46.9 to 51.7)	60.4 (59.3 to 61.4)	52.6 (51.9 to 53.4)	49.8 (49.2 to 50.3)	< 0.001
Marital Status, %					
Married	67.4 (64.6 to 70.1)	36.2 (34.9 to 37.6)	52.9 (51.4 to 54.4)	61.4 (60.4 to 62.4)	< 0.001
Divorced/widowed/separated	13.7 (11.8 to 15.8)	26.9 (25.8 to 28.0)	20.3 (19.4 to 21.3)	22.9 (22.1 to 23.7)	< 0.001
Never married	18.9 (16.8 to 21.2	36.9 (35.7 to 38.1)	26.8 (25.6 to 28.0)	15.7 (15.1 to 16.4)	< 0.001
Education Level, %					
Some college	64.9 (62.1 to 67.7)	42.8 (41.4 to 44.3)	31.1 (29.8 to 32.4)	54.0 (52.9 to 55.1)	< 0.001
High school degree/GED	24.1 (21.7 to 26.7)	43.4 (42.0 to 44.7)	35.8 (34.7 to 37.0)	38.5 (37.6 to 39.4)	< 0.001
No high school degree	10.9 (9.2 to 13.0)	13.8 (12.8 to 14.8)	33.1 (31.7 to 34.5)	7.5 (7.0 to 8.0)	< 0.001
Unemployed, %	33.4 (30.8 to 36.1)	35.8 (34.4 to 37.2)	31.8 (30.3 to 33.3)	36.5 (35.4 to 37.6)	< 0.001
Family Income, %					
> 400% FPL	50.3 (47.1 to 53.5)	27.1 (25.5 to 28.7)	22.7 (21.3 to 24.2)	47.2 (46.0 to 48.5)	< 0.001
251–400% FPL	24.6 (22.2 to 27.2)	30.5 (29.5 to 31.6)	32.2 (30.9 to 33.6)	29.1 (28.3to 29.9)	< 0.001
≤ 250% FPL	25.1 (22.4 to 28.1)	42.4 (40.7 to 44.2)	45.1 (42.9 to 47.2)	23.7 (22.8 to 24.6)	< 0.001
Census Region, %					
Northeast	21.8 (17.7 to 26.4)	14.4 (12.4 to 16.6)	13.4 (11.3 to 15.9)	18.4 (16.8 to 20.1)	< 0.001
Midwest	11.7 (9.2 to 14.8)	17.6 (15.2 to 20.2)	9.0 (7.3 to 11.0)	27.3 (25.7 to 29.0)	< 0.001
South	22.1 (18.2 to 26.7)	59.6 (55.8 to 63.4)	37.8 (32.5 to 43.4)	36.1 (33.9 to 38.2)	< 0.001
West	44.4 (36.6 to 52.5)	8.5 (6.6 to 10.7)	39.8 (35.5 to 44.2)	18.2 (16.7 to 19.8)	< 0.001
Primary Insurance Type, %					
Private	58.4 (55.4 to 61.3)	41.5 (40.0 to 43.0	36.7 (35.0 to 38.5)	52.9 (51.8 to 54.0)	< 0.001
Medicaid	7.2 (5.8 to 8.8)	12.2 (11.2 to 13.1)	10.9 (9.7 to 12.2)	5.1 (4.8 to 5.5)	< 0.001
Medicare	15.4 (13.4 to 17.8)	17.0 (16.1 to 17.9)	11.7 (11.0 to 12.5)	23.4 (22.5 to 24.3)	< 0.001
Other	3.8 (2.9 to 5.0)	5.5 (5.0 to 6.1)	3.4 (3.1 to 3.8)	4.8 (4.5 to 5.1)	< 0.001
Uninsured	15.2 (13.3 to 17.3)	23.8 (22.7 to 24.9)	37.2 (35.4 to 39.1)	13.8 (13.2 to 14.4)	< 0.001
Diabetes, %	19.8 (17.7 to 22.2)	18.6 (17.8 to 19.4)	16.0 (15.2 to 16.7)	15.0 (14.5 to 15.6)	< 0.001
Two Plus Obesity-Related Comorbidities, %	54.3 (51.4 to 57.1)	58.3 (57.1 to 59.5)	46.7 (45.5 to 47.8)	67.2 (66.3 to 68.0)	< 0.001

The data source is MEPS (2011–2016, 2018, and 2020). The study population included adults with a BMI ≥ 30 kg/m^2^ or BMI ≥ 27 kg/m^2^ with one or more weight-related conditions (hypertension, hypercholesterolemia, coronary artery disease, angina, stroke, myocardial infarction, diabetes, asthma, arthritis, history of joint pain, and obesity-related cancer). The adjusted Wald test was used to compare survey-weighted estimates of demographic characteristics across race and ethnicity minority subgroups to that of White individuals. *N* = 91,107

The overall utilization rate of FDA-approved obesity-management medications among all eligible individuals was 1.04%. Liraglutide and phentermine were the most commonly used, accounting for more than 90% of the medications used. The unadjusted utilization rate was 0.34% (95% CI, 0.17% to 0.68%) among Asian individuals, 0.81% (95% CI, 0.65% to 1.00%) among Black individuals, 0.74% (95% CI, 0.57% to 0.95%) among Hispanic individuals, and 1.20% (95% CI, 1.04% to 1.39%) among White individuals (Table 1).

In the main multivariable analysis (Table 2), Asian (aOR, 0.36; 95% CI, 0.16 to 0.77; *P* < 0.01), Black (aOR, 0.51; 95% CI, 0.39 to 0.68; *P* < 0.001), and Hispanic (aOR, 0.70; 95% CI, 0.49 to 0.98; *P* = 0.04) individuals had significantly lower odds of using obesity-management medications compared to White individuals. Higher odds of obesity-management medication use were associated with higher BMI (aOR Class 4 obesity 5.82; 95% CI, 3.32 to 10.20; *P* < 0.001), female gender (aOR, 1.63; 95% CI, 1.31 to 2.03;* P* < 0.001), having diabetes (aOR 5.26; 95% CI, 4.27 to 6.49; *P* < 0.001), and having two or more weight-related conditions (aOR, 1.53; 95% CI, 1.17 to 2.00; *P* < 0.01), while lower odds of utilization were associated with older age (aOR for 75 + years, 0.20; 95% CI, 0.11 to 0.38; *P* < 0.001), lower educational status (aOR for no high school degree, 0.56; 95% CI, 0.34 to 0.93; *P* = 0.03), and unemployment (aOR for unemployment, 0.68; 95% CI, 0.53 to 0.87; *P* < 0.01).

**Table 2:** The Adjusted Association of Race and Ethnicity with Obesity-Management Medication Use Among Medication-Eligible 18 + Year-Olds

	Odds Ratio (95% CI)	*P*-value
Diagnosis		
Overweight + Weight-Related Condition	Reference	N/A
Obesity Class 1	2.48 (1.80 to 3.41)	< 0.001
Obesity Class 2	3.56 (2.54 to 4.98)	< 0.001
Obesity Class 3	4.39 (3.02 to 6.37)	< 0.001
Obesity Class 4	5.82 (3.32 to 10.20)	< 0.001
Age Categories		
18–34 years	Reference	N/A
35–54 years	0.95 (0.44 to 1.35)	0.76
55–64 years	0.63 (0.44 to 0.90)	0.01
65–74 years	0.37 (0.24 to 0.57)	< 0.001
75 + years	0.20 (0.11 to 0.38)	< 0.001
Gender		
Male	Reference	N/A
Female	1.63 (1.31 to 2.03)	< 0.001
Race & Ethnicity		
Asian	0.36 (0.16 to 0.77)	< 0.01
Black	0.51 (0.39 to 0.68)	< 0.001
Hispanic	0.70 (0.49 to 0.98)	0.04
White	Reference	N/A
Marital Status		
Married	Reference	N/A
Divorced/widowed/separated	1.29 (1.01 to 1.66)	0.04
Never married	0.73 (0.57 to 0.95)	0.02
Education Level		
Some college	Reference	N/A
High school degree/GED	0.71 (0.56 to 0.92)	< 0.01
No high school degree	0.56 (0.34 to 0.93)	0.03
Employment Status		
Employed	Reference	N/A
Unemployed	0.68 (0.53 to 0.87)	< 0.01
Family Income		
> 400% FPL	Reference	N/A
251–400% FPL	0.96 (0.74 to 1.25)	0.76
≤ 250% FPL	1.04 (0.79 to 1.36)	0.78
Census Region		
Northeast	Reference	N/A
Midwest	1.43 (0.87to 2.34)	0.15
South	1.73 (1.06 to 2.83)	0.03
West	1.17 (0.69 to 1.98)	0.564
Primary Insurance Type		
Private	Reference	N/A
Medicaid	0.76 (0.51 to 1.14)	0.19
Medicare	1.15 (0.81 to 1.62)	0.44
Other	1.06 (0.65 to 1.74)	0.81
Uninsured	0.64 (0.45 to 0.91)	0.01
Diabetes		
No	Reference	N/A
Yes	5.26 (4.27 to 6.49)	< 0.001
Weight-Related Comorbidities		
0–1 Comorbidities	Reference	N/A
2 + Comorbidities	1.53 (1.17 to 2.00)	< 0.01

The data source is MEPS (2011–2016, 2018, and 2020). The study population included adults with a BMI ≥ 30 kg/m^2^ or BMI ≥ 27 kg/m^2^ with one or more weight-related conditions (hypertension, hypercholesterolemia, coronary artery disease, angina, stroke, myocardial infarction, diabetes, asthma, arthritis, history of joint pain, and obesity-related cancer). The statistical model is a logistic regression model and the estimates given are adjusted odds ratios. 1. *N* = 91,107

The findings from the sensitivity analyses were mostly consistent with the results from the main adjusted analysis. When the study population was expanded to include individuals meeting obesity-management medication utilization criteria using Asian-specific BMI criteria, the likelihood of utilization among Asian individuals decreased by an additional 33% (Table 3) (aOR for Asian ethnicity using Asian-specific BMI criteria 0.24; 95% CI, 0.11 to 0.52; *P* < 0.001 vs. aOR for Asian ethnicity without Asian-specific BMI criteria 0.36; 95% CI, 0.16 to 0.77;* P* < 0.01). In weight-category stratified analyses (Table 4), there was a trend toward lower obesity-management medication use among all racial and ethnic minority groups, relative to White individuals, but only reaching statistical significance in the sub-analysis limited to individuals with class 1 obesity and for Black individuals in the sub-analysis limited to individuals with ≥ class 2 obesity. In the sensitivity analysis expanding the definition of obesity-management medications to include medications for potential off-label use (Supplementary Table 1), statistically significant differences in use remained for all racial and ethnic minority groups.

**Table 3:** The Adjusted Association of Race and Ethnicity with Obesity-Management Medication Use Among Medication-Eligible 18 + Year-Olds Including Asian-specific BMI Thresholds

	Odds Ratio (95% CI)	*P*-value
Diagnosis		
Overweight + Weight-Related Condition	Reference	N/A
Obesity Class 1	2.50 (1.81 to 3.44)	< 0.001
Obesity Class 2	3.59 (2.56 to 5.04)	< 0.001
Obesity Class 3	4.42 (3.04 to 6.44)	< 0.001
Obesity Class 4	5.87 (3.34 to 10.29)	< 0.001
Age Categories		
18–34 years	Reference	N/A
35–54 years	0.95 (0.67 to 1.35)	0.76
55–64 years	0.63 (0.44 to 0.90)	0.01
65–74 years	0.37 (0.24 to 0.58)	< 0.001
75 + years	0.20 (0.11 to 0.38)	< 0.001
Gender		
Male	Reference	N/A
Female	1.63 (1.31 to 2.03)	< 0.001
Race & Ethnicity		
Asian	0.24 (0.11 to 0.52)	< 0.001
Black	0.51 (0.39 to 0.68)	< 0.001
Hispanic	0.70 (0.49 to 0.98)	0.04
White	Reference	N/A
Marital Status		
Married	Reference	N/A
Divorced/widowed/separated	1.29 (1.01 to 1.66)	0.04
Never married	0.73 (0.57 to 0.95)	0.02
Education Level		
Some college	Reference	N/A
High school degree/GED	0.71 (0.56 to 0.92)	< 0.01
No high school degree	0.56 (0.34 to 0.93)	0.03
Employment Status		
Employed	Reference	N/A
Unemployed	0.68 (0.53 to 0.87)	< 0.01
Family Income		
> 400% FPL	Reference	N/A
251–400% FPL	0.96 (0.74 to 1.25)	0.76
≤ 250% FPL	1.04 (0.79 to 1.36)	0.79
Census Region		
Northeast	Reference	N/A
Midwest	1.43 (0.87 to 2.34)	0.15
South	1.73 (1.06 to 2.83)	0.03
West	1.17 (0.69 to 1.98)	0.57
Primary Insurance Type		
Private	Reference	N/A
Medicaid	0.76 (0.51 to 1.14)	0.19
Medicare	1.14 (0.81 to 1.61)	0.44
Other	1.06 (0.65 to 1.74)	0.81
Uninsured	0.64 (0.45 to 0.91)	0.01
Diabetes		
No	Reference	N/A
Yes	5.26 (4.27 to 6.49)	< 0.001
Weight-Related Comorbidities		
0–1 Comorbidities	Reference	N/A
2 + Comorbidities	1.53 (1.17 to 2.00)	< 0.01

The data source is MEPS (2011–2016, 2018, and 2020). The study population was expanded to include individuals meeting criteria for obesity by BMI (BMI ≥ 30 kg/m^2^ and BMI ≥ 27 kg/m^2^ if Asian) and individuals with an elevated BMI (BMI ≥ 27 kg/m^2^ and BMI = 23–26.9 27 kg/m^2^ if Asian) and at least one weight-related condition((hypertension, hypercholesterolemia, coronary artery disease, angina, stroke, myocardial infarction, diabetes, asthma, arthritis, history of joint pain, and obesity-related cancer). The statistical model is a logistic regression model and estimates are given as adjusted odds ratios. *N* = 91,849

**Table 4:** The Adjusted Association of Race and Ethnicity with Obesity-Management Medication Use Among Medication-Eligible 18 + Year-Olds Stratified by Weight Category

	Overweight + Weight-Related Comorbidity		Obesity Class 1		Obesity Class 2 +	
	Odds Ratio (95% CI)	P-value	Odds Ratio (95% CI)	*P*-value	Odds Ratio (95% CI)	*P*-value
Age Categories						
18–34 years	Reference	N/A	Reference	N/A	Reference	N/A
35–54 years	0.60 (0.21 to 1.71)	0.34	1.04 (0.64 to 1.69)	0.89	1.01 (0.63 to 1.62)	0.98
55–64 years	0.30 (0.08 to 1.04)	0.06	0.66 (0.39 to 1.12)	0.12	0.74 (0.45 to 1.20)	0.22
65–74 years	0.20 (0.05 to 0.83)	0.03	0.26 (0.39 to 0.48)	< 0.001	0.57 (0.31 to 1.03)	0.06
75 + years	0.11 (0.02 to 0.57)	< 0.01	0.16 (0.06 to 0.43)	< 0.001	0.26 (0.11 to 0.60)	< 0.01
Gender						
Male	Reference	N/A	Reference	N/A	Reference	N/A
Female	2.29 (1.31 to 4.01)	< 0.01	1.89 (1.38 to 2.58)	< 0.001	1.35 (0.98 to 1.85)	0.07
Race & Ethnicity						
Asian	0.53 (0.16 to 1.81)	0.31	0.28 (0.11 to 0.71)	< 0.01	0.25 (0.05 to 1.20)	0.08
Black	0.74 (0.32 to 1.68)	0.46	0.38 (0.25 to 0.57)	< 0.001	0.58 (0.39 to 0.87)	< 0.01
Hispanic	0.57 (0.24 to 1.38)	0.22	0.62 (0.40 to 0.97)	0.04	0.79 (0.52 to 1.20)	0.27
White	Reference	N/A	Reference	N/A	Reference	N/A
Marital Status						
Married	Reference	N/A	Reference	N/A	Reference	N/A
Divorced/widowed/separated	0.83 (0.36 to 1.94)	0.67	1.38 (0.93 to 2.05)	0.11	1.37 (1.00 to 1.87)	0.05
Never married	0.61 (0.19 to 1.97)	0.41	0.68 (0.42 to 1.09)	0.11	0.81 (0.60 to 1.10)	0.18
Education Level						
Some college	Reference	N/A	Reference	N/A	Reference	N/A
High school degree/GED	0.52 (0.28 to 0.97)	0.04	0.82 (0.61 to 1.12)	0.21	0.69 (0.48 to 0.98)	0.04
No high school degree	0.73 (0.16 to 3.34)	0.69	0.60 (0.31 to 1.17)	0.13	0.50 (0.32 to 0.78)	< 0.01
Employment Status						
Employed	Reference	N/A	Reference	N/A	Reference	N/A
Unemployed	1.15 (0.46 to 2.90)	0.77	0.87 (0.59 to 1.28)	0.48	0.50 (0.37 to 0.66)	< 0.001
Family Income						
> 400% FPL	Reference	N/A	Reference	N/A	Reference	N/A
251–400% FPL	0.86 (0.41 to 1.83)	0.7	0.98 (0.66 to 1.44)	0.91	0.97 (0.65 to 1.46)	0.9
≤ 250% FPL	1.48 (0.69 to 3.17)	0.32	0.84 (0.56 to 1.27)	0.41	1.12 (0.79 to 1.59)	0.52
Census Region						
Northeast	Reference	N/A	Reference	N/A	Reference	N/A
Midwest	1.44 (0.41 to 5.07)	0.57	1.18 (0.69 to 2.03)	0.54	1.68 (0.91 to 3.10)	0.1
South	1.88 (0.57 to 6.15)	0.3	1.54 (0.94 to 2.54)	0.09	1.87 (0.99 to 3.56)	0.06
West	0.87 (0.25 to 2.98)	0.82	1.09 (0.63 to 1.89)	0.76	1.33 (0.69 to 2.58)	0.39
Primary Insurance Type						
Private	Reference	N/A	Reference	N/A	Reference	N/A
Medicaid	0.16 (0.04 to 0.60)	< 0.01	0.62 (0.31 to 1.26)	0.19	1.06 (0.71 to 1.60)	0.77
Medicare	0.69 (0.19 to 2.47)	0.57	1.15 (0.66 to 1.98)	0.62	1.33 (0.86 to 2.05)	0.2
Other	0.47 (0.13 to 1.73)	0.26	1.51 (0.78to 2.93)	0.23	0.81 (0.40 to 1.62)	0.54
Uninsured	0.53 (0.22 to 1.28)	0.16	0.58 (0.35 to 0.96)	0.04	0.72 (0.46 to 1.13)	0.16
Diabetes						
No	Reference	N/A	Reference	N/A	Reference	N/A
Yes	5.85 (3.31 to 10.34)	< 0.001	6.14 (4.25 to 8.87)	< 0.001	4.74 (3.65 to 6.14)	< 0.001
Weight-Related Comorbidities						
0–1 Comorbidities	Reference	N/A	Reference	N/A	Reference	N/A
2 + Comorbidities	1.42 (0.74 to 2.71)	0.29	1.54 (1.03 to 2.31)	0.04	1.53 (0.93 to 2.52)	0.09

The data source is MEPS (2011–2016, 2018, and 2020). The study populations included adults with a BMI 27 kg/m^2^ to 29.9 kg/m^2^ with one or more weight-related conditions (hypertension, hypercholesterolemia, coronary artery disease, angina, stroke, myocardial infarction, diabetes, asthma, arthritis, history of joint pain, and obesity-related cancer) in the in the "Overweight + Weight-Related Comorbidity" category (column 1), adults with a BMI 30 kg/m^2^ to 34.9 kg/m^2^ in the "Obesity Class 1" category (column 2), and adults with a BMI ≥ 35 kg/m^2^ in the "Obesity Class 2 + " category (column 3). The statistical model is a logistic regression model and the estimates given are adjusted odds ratios. 1. *N* = 27,296 2. *N* = 39,654 3. *N* = 24,157

## Discussion

All racial and ethnic groups had lower levels of utilization of obesity-management medication, relative to White individuals. While markers of low socioeconomic status (low education and unemployment) were negatively associated with obesity-management medication use, racial and ethnic differences persisted despite controlling for these factors. Furthermore, racial and ethnic differences in the use of obesity management medications persisted after controlling for having health insurance. The magnitude of the difference in utilization across Asian and White individuals increased when Asian-specific BMI cut-offs were used. These utilization difference trends held across all weight categories and persisted when the obesity-management utilization measure was broadened to include medications for potential off-label use, which supports the overarching hypothesis of this study.

This study makes important contributions to the literature. This is the first study to our knowledge to analyze racial and ethnic differences in obesity-management medication utilization including multiple racial and ethnic groups while considering several potential individual-level socioeconomic confounders. This is the first study that we are aware of to consider the implications of using Asian-specific BMI cut-offs for differences in utilization. This is also the first study to our knowledge to consider the interaction of race and ethnicity with weight category in utilization differences. Other study benefits include the use of survey rather than claims data which allows for inclusion of individuals not receiving health care which would be disproportionately members of racial and ethnic minority groups [27]. Consideration of the role of off-label prescribing in racial and ethnic differences in obesity-management medication use is also a strength [28]. Lastly, the use of survey-based utilization data allows for inclusion of cash-pay prescriptions which would be missed by claims data.

While there are many contributions of this work, it must be interpreted in the context of limitations. Given the cross-sectional nature of the study, no causal claims can be made about the relationship between race, ethnicity, and utilization. Due to data limitations, this study must use BMI to determine utilization eligibility, a flawed measure of adiposity among certain ethnic groups [29]. However, given that BMI is the measure that informs most clinical guidelines, insurance coverage and clinical practice, it is reasonable to use it in this study context [4]. This study may count medications with non-obesity indications as obesity-management medications, potentially leading to an underestimation of differences in utilization given the higher prevalence of diabetes among racial and ethnic minority groups, relative to White individuals. A diabetes indicator is added to the models to minimize this bias. The latest MEPS data for which the BMI measure is available lacks a time frame that reflects the heightened attention that obesity-management medications are receiving in the media which may have implications for utilization differences [30]. Lastly, the medications considered do not include the latest FDA-approved medications for obesity. Despite the limitations of this study, it provides a picture of racial and ethnic differences in utilization that are consistent across a myriad of contexts.

Several factors may underpin the racial and ethnic differences in obesity-management medication utilization observed in the analyses. As expected, racial and ethnic differences in education partially explain differences in obesity-management medication use for Black and Hispanic individuals, underscoring the importance of health literacy for access to care for obesity management in these subgroups [11]. Another unexplored potential contributor to obesity-management medication use differences across race and ethnicity is variability in health insurance quality across race and ethnicity. Specifically, there may be out-of-pocket costs for obesity-management medications faced by insured individuals stemming from plan variation in any coverage, coverage eligibility criteria, and coverage generosity [31, 32]. Out-of-pocket costs may be a salient barrier among Black and Hispanic individuals who have lower socioeconomic status, on average relative to White individuals [33]. This socioeconomic gap is incompletely addressed by the model covariates. Furthermore, many Asian individuals meeting clinical eligibility criteria for obesity-management medication utilization may not meet insurance eligibility criteria for coverage. Insurance criteria may also influence access to medications indirectly by influencing physicians’ perceptions of affordability and subsequent treatment recommendations [34–37]. There may also be differences in patient demand for medications across race and ethnicity stemming from differences in obesity-related concerns, treatment knowledge, and treatment acceptability [38]. However, the only national survey of patients to address obesity-management medications did not find racial or ethnic differences in prevalence of discussions with physicians about this topic or differences in the perceptions of this information as helpful [39]. Nonetheless, there may still be differences in the format of physician communication about these medications across race and ethnicity. Particularly, individuals from racial and ethnic minority groups are less frequently engaged in patient-centered communication, relative to White individuals [40, 41]. Lastly, with respect to Asian individuals, there may be a gap in physician knowledge regarding Asian-specific BMI thresholds [19].

There are steps that have the potential to help reduce racial and ethnic differences in obesity-management medication utilization. In addition to expanding health insurance coverage more broadly and increasing access to primary care, health insurance coverage criteria should reflect obesity-related disease risk by considering additional measures of adiposity [42]. It is also important that clinically-eligible patients be engaged in shared decision-making around obesity-management medication use. Physicians should familiarize themselves with ethnicity-specific BMI thresholds as well as the general cost profile and financial support resources for these medications. Additionally, qualitative work is needed to glean diverse patient perspectives on facilitators and barriers to obesity-management medication use, so culturally-tailored interventions can be developed to help remediate this disparity. A multi-pronged approach is necessary to increase access to obesity-management medications equitably [43]. Lastly, these findings call for the continued exploration of the utilization and perception toward the ever evolving and modernizing obesity medication market. Understanding the perceptions of all populations toward emerging and popularized treatment options is essential to minority health.

## Conclusions

This cross-sectional analysis exploring utilization of obesity-management medications across race and ethnicity found lower levels of utilization among all racial and ethnic minority groups, relative to White individuals. The results of this study suggest that there are disparities in obesity-management medication use. Factors partially accounting for these disparities among Black and Hispanic individuals include lower levels of education, lack of health insurance, and higher levels of public health insurance. With respect to Asian individuals, lower average BMI levels partially explain the disparity in obesity-management medication use.

## Supplementary Information

Below is the link to the electronic supplementary material.

Supplementary file1 (DOCX 16 KB)

## Data Availability

The Medical Expenditure Panel Survey (MEPS) is publically available at https://meps.ahrq.gov/mepsweb/.
