# Supplementary material for: Exploring Racial and Ethnic Differences in Utilization of Medications for Obesity Management in a Nationally Representative Survey
Source: J Racial Ethn Health Disparities. 2024 Dec 17;13(1):329–39. doi: 10.1007/s40615-024-02248-x (PMC12170923; doi:10.1007/s40615-024-02248-x)
Supplement: Supplementary file 1 — Supplementary file1 (DOCX 16 KB) [file 40615_2024_2248_MOESM1_ESM.docx]

| **Supplementary Table 1. The Adjusted Association of Race and Ethnicity with Obesity-Management Medication Use Among Medication-Eligible 18+ Year-Olds Including Use of Off-label Medications for Obesity Management** | | |
| --- | --- | --- |
|  |  |  |
|  | **Odds Ratio (95% CI)** | **P-value** |
| **Diagnosis** | | |
| Overweight + Weight-Related Condition | Reference | N/A |
| Obesity Class 1 | 2.09 (1.64 to 2.67) | <0.001 |
| Obesity Class 2 | 3.03 (2.27 to 4.05) | <0.001 |
| Obesity Class 3 | 3.46 (2.52 to 4.75) | <0.001 |
| Obesity Class 4 | 4.32 (2.55 to 7.29) | <0.001 |
| **Age Categories** |  |  |
| 18-34 years | Reference | N/A |
| 35-54 years | 0.94 (0.67 to 1.32) | 0.71 |
| 55-64 years | 0.72 (0.52to 0.99) | 0.04 |
| 65-74 years | 0.53 (0.35 to 0.80) | <0.01 |
| 75+ years | 0.24 (0.14 to 0.40) | <0.001 |
| **Gender** | | |
| Male | Reference | N/A |
| Female | 1.45 (1.21 to 1.75) | <0.001 |
| **Race & Ethnicity** | | |
| Asian | 0.33 (0.17 to 0.62) | <0.01 |
| Black | 0.53 (0.42 to 0.68) | <0.001 |
| Hispanic | 0.72 (0.54 to 0.95) | 0.02 |
| White | Reference | N/A |
| **Marital Status** | | |
| Married | Reference | N/A |
| Divorced/widowed/separated | 1.15 (0.92 to 1.43) | 0.22 |
| Never married | 0.75 (0.58 to 0.96) | 0.02 |
| **Education Level** | | |
| Some college | Reference | N/A |
| High school degree/GED | 0.74 (0.59 to 0.92) | <0.01 |
| No high school degree | 0.47 (0.30 to 0.72) | <0.01 |
| **Employment Status** | | |
| Employed | Reference | N/A |
| Unemployed | 0.72 (0.57 to 0.90) | <0.01 |
| **Family Income** | | |
| >400% FPL | Reference | N/A |
| 251-400% FPL | 0.99 (0.79 to 1.25) | 0.93 |
| ≤250% FPL | 1.00 (0.80 to 1.24) | 0.99 |
| **Census Region** | | |
| Northeast | Reference | N/A |
| Midwest | 1.18 (0.82 to 1.71) | 0.36 |
| South | 1.36 (0.95 to 1.95) | 0.09 |
| West | 0.99 (0.66 to 1.49) | 0.96 |
| **Primary Insurance Type** | | |
| Private | Reference | N/A |
| Medicaid | 0.74 (0.52 to 1.04) | 0.08 |
| Medicare | 1.03 (0.77 to 1.37) | 0.84 |
| Other | 1.08 (0.71 to 1.62) | 0.73 |
| Uninsured | 0.59 (0.44 to 0.79) | <0.01 |
| **Diabetes** | | |
| No | Reference | N/A |
| Yes | 8.77 (7.22 to 10.65) | <0.001 |
| **Weight-Related Comorbidities** | | |
| 0-1 Comorbidities | Reference | N/A |
| 2+ Comorbidities | 1.58 (1.22 to 2.04) | <0.01 |
| The data source is MEPS (2011-2016, 2018, and 2020). The study population included adults with a BMI ≥30 kg/m2 or BMI ≥27 kg/m2 with one or more weight-related conditions (hypertension, hypercholesterolemia, coronary artery disease, angina, stroke, myocardial infarction, diabetes, asthma, arthritis, history of joint pain, and obesity-related cancer). The utilization measure for this model combined the original measure with off-label GLP-1 receptor agonist (dulaglutide, exanatide, lixisenatide, pramlintide and semaglutide ) and separate components of combination pills prescribed within the same year (Bupropion/Naltrexone, Phentermine/Topiramate). The statistical model is a logistic regression model and the estimates are given adjusted odds ratios. N=91,107 | | |
|  |  |  |
|  |  |  |
